# Student mental health in the midst of the COVID-19 pandemic: A call for further research and immediate solutions

**DOI:** 10.1177/0020764020925108

**Published:** 2020-05-02

**Authors:** Nicholas Grubic, Shaylea Badovinac, Amer M Johri

**Affiliations:** 1 Department of Public Health Sciences, Queen’s University, Kingston, ON, Canada; 2 Department of Psychology, York University, Toronto, ON, Canada; 3 Department of Medicine, Queen’s University, Kingston, ON, Canada

With the global development of the coronavirus disease (COVID-19) outbreak, the psychological issues which accompany this pandemic have rapidly compounded its public health burden (Torales et al., 2020). Emerging research assessing the mental health implications of COVID-19 has identified a heightened prevalence of moderate-to-severe self-reported depressive and anxious symptomatology among the general public (Wang et al., 2020), reflecting the widespread effects of uncertainty and health-related fears. However, further research that investigates beyond the population level is required to understand the individualized disruption of lives and routines as a result of COVID-19, and its associated psychological impacts.

For college students, heightened levels of psychological distress and downstream negative academic consequences are prevalent under normal circumstances (American College Health Association, 2019). As a result of physical distancing measures implemented in response to COVID-19, tertiary education institutions have shifted to an emergency online learning format, which would be expected to further exacerbate academic stressors for students. Based on insights from research examining the impact of academic disruptions on students (Wickens, 2011), it is reasonable to venture that students may experience reduced motivation toward studies, increased pressures to learn independently, abandonment of daily routines, and potentially higher rates of dropout as direct consequences of these measures. Thus, by increasing academic stressors in a population with heightened pre-existing stress levels and a potentially reduced ability to rely on typical coping strategies – such as family who themselves may be experiencing heightened distress – the COVID-19 pandemic has placed an unprecedented mental health burden on students, which urgently requires further examination and immediate intervention.

To date, one published study has explored the impact of COVID-19 on student education and well-being (Cao et al., 2020). Approximately 25% of their sample reported experiencing anxiety symptoms, which were positively correlated with increased concerns about academic delays, economic effects of the pandemic, and impacts on daily life. Furthermore, among the many student surveys administered worldwide, one survey by YoungMinds reported that 83% of young respondents agreed that the pandemic worsened pre-existing mental health conditions, mainly due to school closures, loss of routine, and restricted social connections (YoungMinds, 2020).

These preliminary findings highlight the multiple factors contributing to students’ distress during this pandemic; however, there remains much to be learned about the psychological impacts facing students and what can be done to reduce their negative effects. A timely call to action for further research examining the impact of COVID-19 on student mental health is suggested. Specifically, priorities should include the disturbances to educational progress, adaptations of habitual coping strategies, and approaches academic institutions have taken to reduce adverse academic and psychosocial outcomes. New evidence may help to inform student-centered support programs and mitigate the long-term negative implications for student education and mental health. As we come to terms with the persistent realities of the COVID-19 pandemic, the measures that are taken now to support a vulnerable student population will help mitigate the overall global mental health burden associated with this period of extraordinary disruption and uncertainty.
